# Serum Vitamin D3 as a Potential Biomarker for Neuronal Damage in Smoldering Multiple Sclerosis

**DOI:** 10.3390/ijms251910502

**Published:** 2024-09-29

**Authors:** Natalia Niedziela, Maria Nowak-Kiczmer, Lina Malciene, Mariusz Stasiołek, Jacek T. Niedziela, Zenon P. Czuba, Martyna Lis, Agata Sowa, Monika Adamczyk-Sowa

**Affiliations:** 1 Department of Neurology, Faculty of Medical Sciences in Zabrze, Medical University of Silesia in Katowice, ul. 3-go Maja 13-15, 41-800 Zabrze, Poland; marianowak185@gmail.com (M.N.-K.); agat.sowa@gmail.com (A.S.); m.adamczyk.sowa@gmail.com (M.A.-S.); 2 Klaipeda University Hospital, Lithuanian University of Health Sciences, 44307 Kaunas, Lithuania; lmalciene@gmail.com; 3 Department of Neurology, Medical University of Lodz, ul. Kopcińskiego 22, 90-153 Lodz, Poland; mariusz.stasiolek@umed.lodz.pl; 4 3rd Department of Cardiology, Faculty of Medical Sciences in Zabrze, Medical University of Silesia in Katowice, Silesian Centre for Heart Disease ul, M.C. Sklodowskiej 9, 41-800 Zabrze, Poland; jniedziela@sum.edu.pl; 5 Department of Microbiology and Immunology, Faculty of Medical Sciences in Zabrze, Medical University of Silesia in Katowice, ul. Jordana 19, 41-808 Zabrze, Poland

**Keywords:** vitamin D_3_, GPAF, NF-H, S100B, UCHL1, smoldering multiple sclerosis, neurodegeneration, neuroinflammation

## Abstract

Permanent inflammatory demyelinating and neurodegenerative processes lead to neurological disability in patients with multiple sclerosis (MS). The anti-inflammatory properties of vitamin D_3_ (VitD) are well established, but its role in neurodegeneration is still uncertain. The usefulness of the serum concentration of VitD as a potential biomarker in evaluating brain injury in terms of recently known smoldering MS was under consideration. Methods: We assessed the concentrations of the parameters of brain injury (NF-H, GPAF, S100B, UCHL1) in the cerebrospinal fluid (CSF) of relapsing-remitting (RRMS, *n* = 123) and progressive MS (PMS, *n* = 88) patients in the group with normal levels of VitD (VitDn) and in the VitD deficiency group (VitDd). The levels of NF-H and UCHL1 were higher in the group of VitDd compared to VitDn. The higher serum levels of VitD were correlated with lower concentrations of GFAP, NF-H and S100B in the CSF of the whole group of MS patients and in women with MS as opposed to the levels of UCHL1. In men, there were noted negative correlations between the levels of serum VitD and GFAP and NF-H in CSF but not between VitD and S100B and UCHL1. The negative correlations were observed between VitD and the selected parameters of brain injury in MS patients, in women as well as in men. The concentrations of serum VitD together with selected parameters of brain injury in CSF seem to be promising biomarkers of neurodegeneration processes in smoldering MS.

## 1. Introduction

Multiple sclerosis (MS) is a chronic autoimmune inflammatory neurodegenerative disease characterized by slow deterioration of myelin and axonal transection in the central nervous system (CNS) caused by inflammation [1]. In young adults, MS is a leading cause of non-traumatic disability affecting 2.8 million people worldwide [2], the majority of whom are women [3]. Genetic factors are involved in the risk of MS onset in approximately 25%, while susceptibility to epigenetic, environmental, or gene-environment interactions is also involved [4].

The underlying mechanisms of the etiopathology of MS have not been resolved yet. However, evidence has demonstrated that the disease results from a specific response of the immune system against self-antigens in genetically predisposed individuals combined with the influence of environmental factors [3]. Apart from the geographical latitude and viral infections, vitamin D_3_ (VitD) deficiency is a crucial environmental risk factor in the etiology of MS. Previously, it has been suggested that VitD plays a significant role in the immune regulation in MS [3]. Therefore, there is some evidence about its modulating effect on inflammatory cell differentiation and cytokine secretion [5].

The pathological process in MS results from increased inflammatory responses, destruction of myelin, microglial activation, proliferation of astrocytes, gliosis and neurodegeneration [6,7]. MS is an inflammatory disease based on immune-mediated pathology, in which the pro-inflammatory response from T helper 1 (Th1) and type 17 (Th17) cells is described, thus leading to the damage of myelin and oligodendrocytes [8]. Moreover, axonal injury contributes to the activation of microglial cells, which are the source of cytokines mediating the inflammatory process with subsequent neurodegeneration [9]. Pro-inflammatory interleukins (ILs) increase the permeability of the blood–brain barrier, allowing for demyelination and neurodegeneration in the CNS. The dysregulation of pro- and anti-inflammatory cytokines has been confirmed in MS [3]. Additionally, this imbalance is higher in patients with MS and VitD deficiency [3]. VitD favorably modulates the ratio between pro- and anti-inflammatory ILs. As a result, decreased levels of VitD are a possible cause of the inflammatory process in MS [3]. VitD has anti-inflammatory properties, and low serum concentrations of VitD are associated with the severity of MS pathology as well as increased disability in MS patients [10].

However, the progression of the disease should be seen from a wider perspective, not only as single events but as a dynamic balance between neuroinflammatory and neuroprotective processes [11]. Infiltration of autoreactive B and T lymphocytes is responsible for acute peripheral inflammation, formation of perivascular demyelination and neuroaxonal degeneration. This process manifests as relapses and focal inflammatory lesions in the CNS [12,13]. Simultaneously, resident B cells, macrophages and pro-inflammatory microglia are responsible for chronic neuroinflammation in the CNS [14]. According to the recent concept of smoldering MS, the pathological process of MS is the result of acute peripheral neuroinflammation and chronic neuroinflammation, but focal inflammatory lesions in the CNS are secondary to the loss of axons and neurons from which myelin antigens are released [15]. Impaired recovery processes and inflammatory demyelination result in permanent neurodegeneration and neurological disability in patients with MS [16].

Smoldering MS indicates that the axonal and neuronal loss related to the progressive and irreversible disability is observed from the early stages of the disease [17,18]. Chronic inflammation contributes to an imbalance between damage and the functional reserve of the brain [19]. Higher levels of the parameters of brain injury reflect neuronal damage. Various molecules, including glial fibrillary acidic protein (GFAP), neurofilaments (NFs), ubiquitin C-terminal hydrolase (UCHL1) and glial protein with the calcium-binding domain (S100B), are suspected to be potential biomarkers of neurodegeneration in MS [20]. NFs consist of four subunits: heavy neurofilament (NF-H), medium neurofilament (NF-M), light-chain neurofilament (NF-L) and α-internexin. As the neuronal cytoskeletal proteins, they are abundant in axons [20]. GFAP is an intermediate cytoskeletal protein. It reflects astroglial damage in response to CNS injury because it is released from astrocytes as a molecule primarily expressed in the cytoplasm of astrocytes [21]. UCHL1 is associated with repairing injured axons and neurons. This neuron-specific deubiquitinating enzyme also plays a significant role in immune reactions [22]. S100B is released mainly from astrocytes and is related to neuroinflammation and impairment of axonal conduction [23].

On the other hand, sustained pro-inflammatory cytokine production may reflect the intensity of inflammatory processes and correlate with the progression of the disease [24]. Many studies have indicated immunological properties of VitD by down-regulation of pro-inflammatory ILs, including IL-1, IL-6, IL-17, IFN- γ or TNF-α and simultaneously by up-regulation of anti-inflammatory cytokines, such as IL-4 or IL-10 [5,10,25]. However, the association between VitD and neurodegeneration in the CNS in patients with MS has not yet been investigated.

The aim of the study was to compare the levels of the selected non-standard parameters in the CSF (GFAP, NF-H, S100B, UCHCL1) in patients with MS in the group of VitD deficiency with the group with normal VitD levels. The study was designed to consider the additional usefulness of the serum concentrations of VitD as a potential biomarker in evaluating brain injury in terms of smoldering MS.

## 2. Results

### 2.1. General and Clinical Characteristics of the Study Group

Two hundred and eleven patients with MS were prospectively enrolled in the study. The neurological status of all patients assessed by the Expanded Disability Status Scale (EDSS) score was determined to be 3.50 (2.50, 4.50). The mean age of the whole group was 40 (31–52) years. Women accounted for 64% of the subjects. Patients on disease-modifying therapy (DMT) at the time of the study constituted 12.0% of the study group. Median VitD concentration [ng/mL] in all MS individuals was 26 (20–37), 25 (24–40) and 28 (18–36) in men and women, respectively. VitD deficiency was found in 61% of them. Active lesions on brain and spinal cord magnetic resonance imaging (MRI) were reported in 27% and 9.5% of the whole study group, respectively. Concomitant diseases, including arterial hypertension (HA), dyslipidemia, overweight or obesity, diabetes mellitus and thyroid function abnormalities, were noted in 26%, 66%, 34%, 4.3% and 8.5% in all MS patients, respectively.

The whole study group included 123 patients with relapsing-remitting (RRMS) and 88 subjects with progressive MS (PMS) (56 with primary progressive MS [PPMS] and 32 with secondary progressive MS [SPMS]). The general and clinical characteristics of the study groups are given in Table 1. The proportion of females with RRMS was higher than in the PMS group. RRMS patients experienced more frequent relapses, thyroid function abnormalities, diabetes mellitus and elevated high-density lipoprotein (HDL) and had more frequent Gd+ lesions on brain MRIs compared to the PMS group. Patients with PMS were older than those with RRMS and had higher total scores measured by the EDSS, longer MS duration and longer duration of MS symptoms compared to the subjects with RRMS. Significant differences were also observed in HA and in the concentrations of total cholesterol, low-density lipoprotein (LDL) and triglycerides (TGs). No differences were found regarding overweight, obesity, smoking, serum VitD concentrations, VitD deficiency, oligoclonal bands (OCBs) or elevated IgG in the CSF between RRMS and PMS patients.

#### 2.1.1. General and Clinical Characteristics of the Groups with VitD Deficiency and Normal VitD Levels

Among all MS patients, the serum concentration of VitD was evaluated in 208 subjects. There were 82 patients with normal VitD (VitDn) levels and 126 subjects with VitD deficiency (VitDd). The general and clinical characteristics of the VitDn and VitDd groups are given in Table 2. Patients from the VitDn group were older and had longer MS duration and a higher EDSS score compared to the VitDd group. The proportion of patients on DMT was higher in VitDn compared to VitDd. The patients from the VitDd group had higher levels of total cholesterol and LDL than those from the VitDn group. Significant differences were found in terms of concomitant diseases, including dyslipidemia, HA, diabetes mellitus and smoking in the VitDd group compared to the VitDn group.

#### 2.1.2. Assessment of the Selected Parameters of Brain Injury in the CSF in the Groups with VitD Deficiency and Normal VitD Levels

The concentrations of NF-H and UCHL1 in the CSF were higher in the VitDd group compared to the VitDn group, while the levels of GFAP and S100B did not differ between the groups. The comparison of the selected parameters of brain injury in the CSF in the groups with VitD deficiency and normal VitD levels is given in Table 3.

#### 2.1.3. Correlations between the Serum VitD Concentration and the Selected Parameters of Brain Injury in CSF in the Study MS Group Depending on Sex

**Figure 1:**
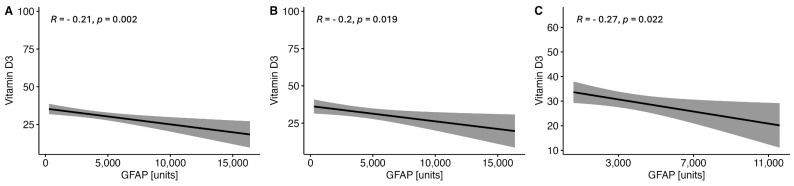
Correlations between the serum VitD and GFAP concentrations in the whole study MS group (**A**) and subgroups of women (**B**) and men (**C**).

**Figure 2:**
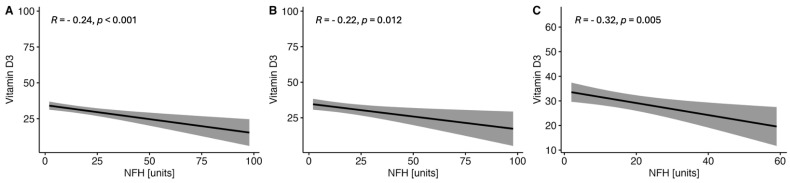
Correlations between the serum VitD and NF-H concentrations in the whole study MS group (**A**) and subgroups of women (**B**) and men (**C**).

**Figure 3:**
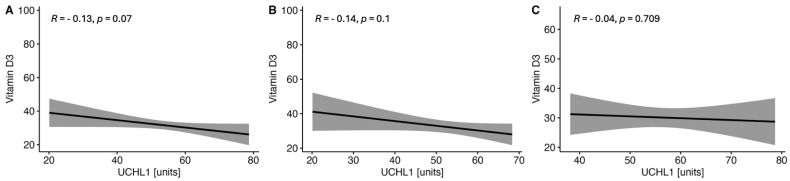
Correlations between the serum VitD and UCHL1 concentrations in the whole study MS group (**A**) and subgroups of women (**B**) and men (**C**).

**Figure 4:**
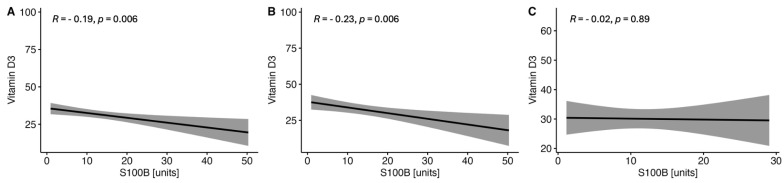
Correlations between the serum VitD and S100B concentrations in the whole study MS group (**A**) and subgroups of women (**B**) and men (**C**).

There were observed negative correlations between the levels of serum VitD and GFAP (Figure 1) and NF-H (Figure 2) in the whole MS group and in both women and men subgroups. On the other hand, no correlations were found between the serum VitD and UCHCL1 concentration in CSF in the MS group and among women and men (Figure 3). The negative correlations between serum VitD and S100B levels were observed in the whole MS group and only in the women subgroup, while no such correlation was found among men (Figure 4).

## 3. Discussion

Recently, increased attention has been paid to the role of VitD apart from calcium homeostasis and bone health in different areas of medical research. VitD hydroxylation enzymes and its receptors are located in many internal organs, immune cells and some parts of the brain [26]. Vitamin D is primarily obtained via sun exposure by ultraviolet (UV) B radiation, converting 7-dehydrocholesterol to cholecalciferol. The first hydroxylation of cholecalciferol leads to the 25-hydroxyvitamin (25(OH)D with subsequent hydroxylation and production of 1,25-dihydroxy vitamin D (1,25(OH)2D [27].

The exact etiology of MS is complex, based on different pathologic mechanisms connected with altered immune function, genetics and environmental factors. Several epidemiological studies have reported a link between low VitD concentrations and an increased risk of MS [28,29]. VitDd is a significant risk factor for the development of MS in childhood and adolescence [29]. In the context of increasing latitude, there is some evidence for an earlier onset and higher MS prevalence with the increasing distance from the equator and decreasing annual sunlight exposure [27]. Furthermore, the dietary source of VitD and consumption of fatty fish can play a protective role [27,30]. Additionally, a genetic association between HLA-DRB1*1501 and VitD regulation was reported [27].

On the other hand, lower serum levels of VitD not only are associated with an increased risk of the development of MS but also contribute to the progression rate and disease severity in early MS or clinically isolated syndromes (CIS) [28]. Some evidence suggested that higher serum concentrations of VitD were connected with a more favorable disease course with less MRI activity and fewer relapses [26]. Several studies consistently indicated an inverse relationship between the levels of VitD and disability progression, relapse rate and the occurrence of new lesions on MRI [26,31].

VitD with its anti-inflammatory properties plays an important role in the immune response through down-regulation of pro-inflammatory cytokines [10]. Apart from the classic role of VitD in calcium and phosphate homeostasis, VitD is also involved in cell proliferation and differentiation. It acts as an immunomodulator, regulating the secretion of different cytokines and shifting the immune response towards a less inflammatory profile. Additionally, it promotes the expression of regulatory T lymphocytes (Treg) and growth factors and influences cell signaling and the response to antioxidant effect, as a results contributing to the protection of neuroinflammation [32,33,34]. The mechanism of reducing inflammation by VitD-mediated immunological activity is well established. However, evidence of its role in neurodegeneration and its potential neuroprotective effects is still lacking.

Meanwhile, as regards smoldering MS, the disease is not focal inflammation of the CNS, but there is a diffuse smoldering pathophysiological process that occurs with concomitant inflammation [15]. The infiltrating autoreactive B and T lymphocytes are responsible for acute peripheral inflammation, formation of perivascular demyelination and neuroaxonal degeneration, which manifests as relapses and focal inflammation in the CNS [12,13]. At the same time, macrophages, resident B cells and pro-inflammatory microglia contribute to chronic neuroinflammation in the CSF. In smoldering MS, acute peripheral neuroinflammation and chronic neuroinflammation co-exist, but focal inflammatory lesions in the CNS are secondary to the loss of axons and neurons from which myelin antigens are released [15]. Some insights show that VitD counteracts neurodegeneration and oxidative stress by inhibiting reactive M1 microglia and astrocytes. Both active forms of VitD 25(OH)D and 1,25(OH)2D cross the blood–brain barrier (BBB) and activate various glial and neuronal cells [27]. Moreover, the enzyme responsible for converting 25(OH)D to 1,25(OH)2D is expressed in neurons, astrocytes and microglia [27,35]. Furthermore, VitD has been suspected of protecting microglia and macrophage activation and of protecting cuprizone-induced demyelination [10].

Permanent inflammatory demyelination and impaired repair process in smoldering MS result in neurodegeneration and neurological disability in patients with MS. A clinical indicator of smoldering MS is progression independent of relapse activity (PIRA), which needs to be distinguished from relapse-associated worsening (RAW). It is confirmed that PIRA and RAW are common processes leading to confirmed disability accumulation (CDA) in relapsing and progressive phenotypes of MS [36]. The mechanism of VitDd affects neurodegeneration, myelination and immune regulation. It is crucial for increasing progression in MS patients. To the best of our knowledge, our study for the first time analyzed the association between neuronal damage reflected by the levels of parameters of brain injury in the CSF and the serum concentrations of VitD as the potential modulator of neurodegeneration in MS.

There are some molecules, including GFAP, NFs, C-terminal hydrolase, UCHL1 and S100B, that are recognized as indicators of neuronal damage and suspected to be potential biomarkers of neurodegeneration in MS. Compared to serum, the CSF is closer to the brain and contains higher levels of CNS-derived proteins. Potentially, it represents better glial and neuronal biomarkers reflecting brain injury in MS [37]. In our study, we confirmed a negative correlation between the serum levels of VitD and the concentrations of GFAP, NF-H and S100B in the CSF patients with MS, which suggests that lower levels of VitD play an unfavorable role in brain injury reflected by higher concentrations of biomarkers of neurodegeneration in the CSF. These results underline that adequate serum VitD concentrations are not only crucial for suppressing inflammation and reducing neurodegeneration in MS patients. Considering that in smoldering MS, irreversible and progressive disability caused by axonal and neuronal loss is found in the early stages of the disease, the additional neuroprotective role of higher serum levels of VitD is promising.

GFAP is rapidly released in axonal degeneration due to astrogliosis. Therefore, it has emerged as a CSF biomarker of brain injury [38]. Previously, in their meta-analysis, Momtazmanesh et al. showed higher GFAP in the CSF of PPMS patients compared to RRMS subjects [20]. In another meta-analysis, including 11 clinical trials comprising 960 MS patients, GFAP concentrations in the CSF were significantly elevated in MS patients compared to the healthy controls, and the mean levels of GFAP in the CSF were higher in PMS subjects compared to RRMS patients [39]. The levels of GFAP in the CSF reflect different degrees of damage to astrocytes in different MS phenotypes, which facilitates MS diagnosis and distinguishes the subtypes of MS. In our study, we included patients with RRMS and PMS, but no differences in VitD concentrations or VitDd were observed between the groups. Whether the level of VitD differs among the different phenotypes of MS is a matter of debate, with some studies reporting higher values in RRMS compared to PMS [40] as opposed to those indicating no differences [41]. Additionally, at the time of MS diagnosis, low VitD status may be connected with an earlier conversion to SPMS [42]. Currently, there are no studies showing the association between the serum levels of VitD and GFAP in the CSF of MS patients and various phenotypes of the disease. However, Scrimgeour et al. reported that VitD intake decreased GFAP and UCHL-1 in experimental traumatic brain injury [43], which confirmed the necessity for further investigation of this issue.

NFs are intermediate filament neuronal cytoskeletal proteins released as a response to neuroaxonal injury. In their meta-analysis, Momtazmanesh et al. reported that patients with CIS and MS had higher NF-L levels in the CSF compared to the controls. However, no differences were found between RRMS and PMS groups [20]. In turn, Martin et al. showed higher levels of NF-L in the CSF of RRMS patients compared to PMS subjects [44]. In another study, NF-L in the CSF increased six years before the clinical MS onset [45]. In our study, NF-H in the CSF correlated negatively with the serum levels of VitD. Our result was in line with the findings of Sandberg et al., who showed an inverse association between serum VitD and the levels of NF-L in the CSF of patients with MS and suggested that high concentrations of VitD were connected with decreased axonal injury in MS [46]. Weekly supplementation with 20,000 IU of VitD decreased NF-L levels in the serum of MS patients who were not on DMT [47]. On the other hand, Rosjo et al. did not confirm associations between serum levels of NF-L and VitD independently of interferon-beta therapy in patients with RRMS [48].

Similarly, supplementation of high-dose of VitD for 48 weeks was not connected with lower serum NF-L levels in RRMS subjects [49]. Additionally, an increase in serum VitD can reduce MRI activity in VitD-sufficient RRMS patients [50]. At the same time, there were higher serum NF-L levels in patients with MRI disease activity compared to MRI without Gd+ lesions [48]. In our study, we found a higher concentration of NF-H in the CSF in the group of patients with VitDd compared to the VitDn group. Similarly, in the animal model of PMS, the animals supplemented with VitD had lower serum NF-L levels. VitD prevented oxidative damage and neurodegeneration as well as enhanced remyelination [51]. Moreover, it was suggested that DMT could modify the levels of NF-L. Treatment with natalizumab in the EXPAND trial or siponimod in the ASCEND clinical trial caused a reduction in serum NF-L concentrations [52].

To conclude, NFs seem to be promising predictors for distinguishing MS patients at the early stage of the disorder, conversion of CIS to MS, or disease activity. It could also be a potential prognostic marker of DMT effectiveness. However, there are many discrepancies related to the action of NFs, and the difference could be explained by confounding factors, including age, sex, DMT, or the evaluation of various subunits or NFs in different body fluids. Some data indicated that age could be a vital determinant of the levels of NFL and GFAP in the CSF [20]. Nevertheless, in our study, the level of NF-H was higher in the VitDd group that was younger than VitDn patients. Based on our insights and previous studies, the application of NFs for detecting neurodegeneration and PIRA in smoldering MS in connection with the serum concentration of VitD in patients with MS requires further analysis.

Ca2+ binding protein S100B is another molecule highly expressed in the CNS after brain injury. The physiological concentrations of S100B lead to cell proliferation and migration as well as neurite outgrowth and synaptogenesis. In the case of higher levels of S100B, microglial activation and exacerbation of the inflammatory response by releasing proinflammatory cytokines and stress-related enzymes were reported [53]. In the meta-analysis, the concentration of S100B was significantly higher in the CSF of patients with MS compared to the controls [20]. Barateiro et al. showed increased S100B levels at the time of MS diagnosis [54]. However, they found no differences between the concentrations of S100B in the CSF in RRMS and SPMS subjects [55]. In our study, we indicated that higher levels of serum VitD were associated with lower concentrations of S100B in the CSF of patients with MS. No studies have evaluated the association between ViD and S100B in MS. Considering that elevated S100B levels reflect brain injury, higher levels of serum VitD are crucial for reducing neurodegeneration in the CNS. On the other hand, chronic active lesions characterizing smoldering MS showed elevated S100B levels in demyelinated areas with the lower expression of its receptor in the rim [23]. Chronic active lesions are reflected on MRI by slowly expanding lesions (SELs), which should also be assessed according to different levels of serum VitD.

UCHL plays a significant role in immune reactions and the repair of injured axons. As a small molecule expressed in neurons and neuroendocrine cells, UCHL plays a significant role in the redox state and degradation and removal of selected proteins. Loss of UCHL1 function due to reactive oxygen species, NO, or reactive lipids is related to the pathogenesis of brain injury and neurodegeneration [56]. The concentration of UCHL1 was increased in serum and the CSF after traumatic brain injury, and it was associated with its severity and long-term outcome [22,57]. In our study, we found higher levels of UCHL1 in the VitDd group compared to the VitDn group. However, to date, the role of UCHL1 in MS has hardly been investigated. Górska et al. showed higher UCHL1 concentrations in the plasma of RRMS patients compared to healthy individuals. Simultaneously, the level of UCHL1 did not correlate with the number of relapses during 24 months, EDSS, the number of years from the first symptoms to MS diagnosis or the age of the patients [22]. Nevertheless, the level of VitD together with UCHL may reflect neurodegeneration in MS. However, more detailed knowledge about the distribution of UCHL1 in the CNS and plasma is required.

The EDSS score is a well-established method for evaluating the degree of disability and monitoring patients with MS [58]. Surprisingly, in our study, patients in the VitDn group were assessed as having higher EDSS scores compared to the VitDd group. The explanation is not clear. A higher EDSS was most likely related to more advanced age and a longer duration of MS in the VitDn group. Patients enrolled in our study did not have VitD supplementation for three months prior to the inclusion. However, older patients might have used VitD more often in the past. Therefore, the beneficial effect of VitD for disability in patients with MS may be long-term. On the other hand, the prognostic value of serum levels of VitD for the progression of disability on EDSS is uncertain. Higher concentrations of serum VitD were connected with lower EDSS scores in patients with CIS [59]. Some studies showed that low concentrations of VitD could be associated with increased disability on EDSS in MS patients [40], but this association was not always found [10]. Furthermore, as regards smoldering MS, routine neurological examination of patients according to the EDSS does not seem to be sufficiently sensitive to find neuronal damage resulting in disability progression. Cadavid et al. suggested the use of the EDSS-Plus, additionally including the Timed 25-Foot Walk test (T25FW) and the 9-Hole Peg Test (9HPT), which clearly separated SPMS progressors from non-progressors [60].

Many different environmental and non-environmental factors can modulate the levels of serum VitD. Sex is one of the potential factors that may influence VitD concentration. Significant differences in the levels of VitD between sexes were reported. Moreover, sex hormones, including estrogens and androgens, affect the expression of genes related to VitD. In addition, estradiol increased the expression of the VitD receptor gene in the CNS. There were also some differences between VitD metabolism in males and females with MS [61]. In our study, negative correlations were found between serum VitD and the parameters of brain injury in the CSF of MS patients in women as well as in men. Women had a greater ability to accumulate the active form of VitD. Therefore, its potential role in the immune response and neurodegeneration should be stronger in female MS patients. Nevertheless, the same results in men and women confirmed that higher levels of VitD could be protective for neurodegeneration in MS patients irrespective of sex.

Serum VitD levels can change under various clinical conditions. Among crucial aspects influencing the status of VitD, lower physical activity, obesity [62], smoking, ethnicity, age, UVB radiation, sex and genetic or individual metabolism of VitD should be mentioned [61]. We found a higher proportion of dyslipidemia, HA, diabetes mellitus and smoking in the VitDd group compared to VitDn patients. It indicates a significant and complex role of Vitd. It seems unclear whether co-existing diseases can modify the rate of neuronal damage in relation to the different levels of serum VitD. A better understanding of these interactions may lead to dietary recommendations and guidelines for VitD supplementation in MS patients.

So far, many interventional studies have been performed to investigate whether VitD supplementation can have a beneficial effect on the disease course and progression, but study designs and methodology have not always been appropriate [26]. The supplementation of VitD in clinical trials was related to MS activity to a lesser extent than that reported in observational research. This may be due to various inclusion criteria, the dose of VitD or the duration of supplementation. There were different doses of VitD in patients with various DMTs. However, two randomized clinical trials (CHOLINE and SOLAR) concluded that the number of new or enlarging T2 lesions, new T1 lesions, annualized relapse rate and disease progression decreased after VitD supplementation [50].

Additionally, it was found that a daily dose of 10,400 IU mediated pleiotropic immunomodulatory functions by reducing memory effector CD4+ T cells and decreased secretion of IL-17. This dose was safe and tolerable for patients with MS [63]. The impact of VitD supplementation on the parameters of brain injury in the CSF has not been determined yet. Much evidence indicates less active MS in the presence of higher serum concentrations of VitD. Nevertheless, how much VitD patients should take to reduce neurodegeneration in smoldering MS remains unknown.

The study has some limitations. Firstly, the patients enrolled in the study used DMT or were not treated. Additionally, they used different treatment regimens. Based on our knowledge, some types of DMT may be related to the decrease in the concentrations of the brain injury parameters. Furthermore, the effect of VitD may also be influenced by DMT that patients are given. Secondly, we performed the analysis from October 2023 to March 2024 in the same climate zone and latitude to eliminate the differences in insolation, but the accurate daily sun exposure was not considered in the context of VitD synthesis via UVB radiation.

Similarly, dietary intake should be determined to assess the serum levels of VitD more adequately. Unfortunately, to date, studies on the assessment of the parameters of brain injury in the CSF of patients with MS have been limited and together with serum levels of VitD have been hardly investigated. Therefore, the appropriate discussion and comparison of our results with the findings of other authors was difficult.

### Future Directions

Future studies are warranted to further investigate applications of neuronal and glial biomarkers and VitD in the clinical practice of MS patients. Some analyses reported the role of brain injury parameters in distinguishing patients with MS from the controls. Meanwhile, determining the value of brain injury biomarkers in connection with the serum levels of VitD to facilitate MS diagnosis, differentiate various subtypes and evaluate neuronal damage and disease progression in terms of smoldering MS seems to be most desirable. Moreover, patients of ethnicities other than European and of different latitudes should also be included in the analysis. The results should be validated in a larger group of patients using various measurement methods to assess brain injury parameters, taking into account serum and CSF assessment and new radiological parameters of neuroimaging in smoldering MS. To prevent neurodegeneration, recommendations related to the adequate doses and duration of VitD supplementation in patients with MS are necessary.

## 4. Materials and Methods

All consecutive patients with MS were prospectively enrolled from the Department of Neurology in Zabrze, Medical University of Silesia, Katowice, Poland. The study was performed from October 2023 to March 2024. Depending on serum VitD concentrations, all patients were divided into two groups: VitDn (serum VitD level > 30 ng/mL, >75 nmoL/L) and VitDd (serum VitD level < 30 ng/mL, <75 nmoL/L). Additionally, the analyses were performed in the subgroups related to the clinical phenotype of MS—RRMS and PMS (PPMS/SPMS) groups.

The inclusion criteria were as follows: written informed consent for participation in the study, Caucasian race, residence in the territory of the Upper Silesia, RRMS or PPMS diagnosed according to the McDonald criteria (2017), and in the case of SPMS, the following: evidence of progression ≥ 3 months, disability progression by 1 step on EDSS in patients with EDSS ≤ 5.5 or 0.5 EDSS steps in patients with EDSS ≥ 6.0, age ≥ 18 years, no immunomodulatory treatment before the study or DMT, EDSS ≤ 5.0 (RRMS and PPMS), EDSS ≥ 4.0 and pyramidal functional system (FS) 2.0 (SPMS) [64]. The exclusion criteria were as follows: VitD supplementation for three months prior to the study, indoor stay only or underground work, traveling to another climate zone < 6 months before the study, relapse and steroid therapy during the last six months, contraindications for MRI and lumbar puncture, neurodegenerative diseases other than MS or other neurological or severe illness that could affect the neurological examination, intestinal malabsorption syndromes, history of head injury and stroke during one year before the study, other severe autoimmune disorders, pregnancy and breastfeeding.

The concentrations of the parameters of brain injury, including NF-H, GFAP, S100B and UCHL1, were evaluated in the CSF using Invitrogen Brain Injury 4-plex Human (Carlsbad, CA, USA). All steps of the analysis were performed according to the manufacturer’s instructions. Fasting serum samples collected between 7 a.m. and 8 a.m. were used for biochemical tests. The 25(OH)-Vitamin D direct day enzyme-linked immunosorbent assay (ELISA) Kit (Immundiagnostik AG, Bensheim, Germany) was used to determine the level of 25-hydroxycholecalciferol [25(OH)D]. The range of reference values was as follows: normal level (>30 ng/mL; >75 nmoL/L) and VitD deficiency (<30 ng/mL; <75 nmoL/L). The abbreviation 25(OH)D was synonymous with vitamin D_3_ and VitD and was used interchangeably.

All diagnostic procedures were performed during the morning medical visit and included physical examination, medical history, the panel of biochemistry blood tests and lumbar puncture. The clinical stage of MS was determined using the EDSS assessed by an experienced EDSS rater. Patient medical histories were analyzed for MS, treatment and comorbidities. The survey questionnaire was prepared to provide additional data related to basic personal data, VitD supplementation, underlying disease, relapses, DMT use and steroid therapy or lifestyle. Lumbar puncture (L3/L4 or L4/L5) was typically performed to collect the CSF for further analysis. All patients underwent MRI examination of the brain and cervical and thoracic spine. The presence of Gd+ lesions was assessed.

The study was approved by the Bioethics Committee of the Medical University of Silesia in Katowice (consent no. PCN/022/KB1/48/I/20).

### Statistical Analysis

Descriptive statistical parameters for continuous variables were presented as median and interquartile range. Qualitative variables were presented as percentage values. The Mann–Whitney U test was used to compare two subgroups. Group homogeneity with respect to the qualitative variables was analyzed by the chi-squared test using the Fisher’s exact test when the expected frequency table included the values below five. The frequencies between the subgroups were compared using the contingency tables and the chi-square test. The linear correlations between variables were calculated using the Pearson’s R test for correlation. The significance level of *p* < 0.05 was adopted. All statistical analyses were performed using R version 4.2.2 (R Core Team (2022); R: A language and environment for statistical computing; R Foundation for Statistical Computing, Vienna, Austria) and RStudio (RStudio Team (2020); RStudio: Integrated Development for R. RStudio, PBC, Boston, MA, USA).

## 5. Conclusions

To conclude, the results of our study performed in the prospectively recruited group of patients with MS have indicated that GFAP, NF-H, S100B and UCHL1 are promising biomarkers of neurodegeneration and neuroinflammation in MS.

Most patients with MS presented with VitDd. We found higher concentrations of NF-H and UCHL1 with co-existing diseases in the VitDd subjects compared to the VitDn group. Higher serum levels of VitD were correlated with lower concentrations of GFAP, NF-H and S100B in the CSF of MS patients. Negative correlations between VitD and the selected parameters of brain injury were observed in women and men.

Based on the concept of smoldering MS, axonal and neuronal loss is found in the early stages of the disease, leading to progressive and irreversible disability in MS patients with a further suggestion of a continuum of relapsing and progressive phenotypes of MS. Based on this concept, the early detection of neurodegeneration using brain injury parameters is crucial to prevent disability in patients with MS. Some analyses reported the role of brain injury parameters to distinguish patients with MS from the control individuals or to describe disease course. Meanwhile, determining the value of brain injury biomarkers in connection with the serum levels of VitD to make MS diagnosis easier, differentiate various subtypes and evaluate neuronal damage and disease progression in terms smoldering MS seems to be most desirable.

Moreover, patients of other than European ethnicity and latitude may also be enrolled in the analysis. The results should be validated in larger groups of patients using various measurement techniques of brain injury parameters, taking into account serum and CSF analysis and new radiological parameters of neuroimaging in smoldering MS. To prevent neurodegeneration, recommendations related to adequate doses and duration of VitD supplementation in patients with MS are necessary.

To the best of our knowledge, our study is the first to analyze the associations between the levels of serum VitD and brain injury parameters in the CSF in patients with MS. Our results confirmed that VitD with its well-known anti-inflammatory properties also demonstrated the chance to be a potential biomarker in the evaluation of brain injury in terms of smoldering MS. Further studies are warranted in this respect.

## Figures and Tables

**Table 1:** General and clinical characteristics of the study group.

Parameter	RRMS (n = 123)	PMS (n = 88)	*p*-Value
Age (years)	36 (29–41)	52 (47–54)	<0.001
Sex (females)	87/123 (71%)	48/88 (55%)	0.016
MS relapse	30/123 (24%)	0/88 (0%)	<0.001
MS duration (years)	0 (0–1)	1 (0–12)	<0.001
MS symptoms duration (years)	1 (0–3)	5 (1–13)	<0.001
EDSS (score)	2.50 (2.00–3.00)	4.50 (4.00–5.00)	<0.001
DMT	18/123 (15%)	8/88 (9.1%)	0.227
Gd+ lesions (brain MRI)	48/123 (39%)	8/88 (9.1%)	<0.001
Gd+ lesions (cervical and thoracic MRI)	12/123 (9.8%)	8/88 (9.1%)	0.871
Elevated IgG in the CSF	75/105 (71%)	64/88 (73%)	0.841
OCBs	78/117 (67%)	48/88 (55%)	0.078
Vitamin D3 deficiency	78/120 (65%)	48/88 (55%)	0.127
Vitamin D3 concentration (ng/mL)	27 (18–36)	26 (21–51)	0.131
Dyslipidemia	75/123 (61%)	64/88 (73%)	0.076
Total cholesterol (mmoL/L)	5.00 (4.29–5.55)	6.21 (4.15–6.38)	<0.001
LDL cholesterol (mmoL/L)	2.75 (2.23–3.37)	3.65 (2.43–4.19)	<0.001
HDL cholesterol (mmoL/L)	1.56 (1.30–2.00)	1.41 (1.09–1.66)	0.002
Triglycerides (mmoL/L)	1.11 (0.78–1.43)	1.36 (1.18–1.98)	<0.001
Overweight or obesity	39/123 (32%)	32/88 (36%)	0.480
Hypertension	15/123 (12%)	40/88 (45%)	<0.001
Diabetes mellitus	9/123 (7.3%)	0/88 (0%)	0.011
Thyroid function abnormalities	18/123 (15%)	0/88 (0%)	<0.001
Smoking	12/123 (9.8%)	16/88 (18%)	0.075

EDSS—Expanded Disability Status Scale; RRMS—relapsing-remitting multiple sclerosis; PMS—progressive multiple sclerosis; MS—multiple sclerosis; DMT—disease-modifying therapy; Gd+—gadolinium-enhancing lesions; MRI—magnetic resonance imaging; CSF—cerebrospinal fluid; OCBs—oligoclonal bands; LDL—low-density lipoprotein; HDL—high-density lipoprotein.

**Table 2:** General and clinical characteristics of the groups with VitD deficiency and normal VitD levels.

Parameter	Overall n = 208	VitDn n = 82	VitDd n = 126	*p*-Value
SM group				0.127
RRMS	120/208 (58%)	42/82 (51%)	78/126 (62%)	
PMS	88/208 (42%)	40/82 (49%)	48/126 (38%)	
Age (years)	41 (31, 52)	48 (39, 52)	37 (29, 48)	<0.001
Sex (females)	135/208 (65%)	59/82 (72%)	76/126 (60%)	0.086
SM relapse	30/208 (14%)	15/82 (18%)	15/126 (12%)	0.200
SM duration [years]	0 (0, 1)	1 (0, 12)	0 (0, 1)	0.004
Duration of SM symptoms [years]	2 (0, 6)	2 (0, 14)	2 (1, 5)	0.442
EDSS (score)	3.50 (2.50, 4.50)	3.75 (3.00, 5.00)	3.25 (2.00, 4.00)	<0.001
DMT	26/208 (13%)	23/82 (28%)	3/126 (2.4%)	<0.001
Gd+ lesions (brain MRI)	56/208 (27%)	18/82 (22%)	38/126 (30%)	0.192
Gd+ lesions (cervical and thoracic MRI)	20/208 (9.6%)	6/82 (7.3%)	14/126 (11%)	0.364
Elevated IgG in the CSF	136/190 (72%)	57/79 (72%)	79/111 (71%)	0.883
OCBs	123/202 (61%)	48/82 (59%)	75/120 (63%)	0.571
Vitamin D3 (ng/mL)	26 (20, 37)	41 (34, 54)	22 (17, 25)	<0.001
Dyslipidemia	136/208 (65%)	62/82 (76%)	74/126 (59%)	0.012
Total cholesterol (mmoL/L)	5.29 (4.23, 6.16)	5.77 (4.76, 6.33)	4.76 (4.22, 5.66)	0.011
LDL cholesterol (mmoL/L)	2.75 (2.36, 3.65)	3.42 (2.47, 3.92)	2.72 (2.30, 3.44)	0.004
HDL cholesterol (mmoL/L)	1.51 (1.15, 1.79)	1.51 (1.15, 1.89)	1.56 (1.23, 1.76)	0.799
Triglycerides (mmoL/L)	1.20 (0.86, 1.64)	1.26 (0.89, 1.38)	1.20 (0.89, 1.98)	0.194
Overweight or obesity	71/208 (34%)	28/82 (34%)	43/126 (34%)	0.998
Hypertension	55/208 (26%)	35/82 (43%)	20/126 (16%)	<0.001
Diabetes mellitus	9/208 (4.3%)	0/82 (0%)	9/126 (7.1%)	0.013
Thyroid function abnormalities	18/208 (8.7%)	12/82 (15%)	6/126 (4.8%)	0.013
Smoking	28/208 (13%)	3/82 (3.7%)	25/126 (20%)	<0.001

VitDn—the group with the normal vitamin D3 levels; VitDd—the group with vitamin D3 deficiency; EDSS—Expanded Disability Status Scale; RRMS—relapsing-remitting multiple sclerosis; PMS—progressive multiple sclerosis; MS—multiple sclerosis; DMT—disease-modifying therapy; Gd+—gadolinium-enhancing lesions; MRI—magnetic resonance imaging; CSF—cerebrospinal fluid; OCBs—oligoclonal bands; LDL—low-density lipoprotein; HDL—high-density lipoprotein.

**Table 3:** The comparison of the selected parameters of brain injury in the CSF in the groups with VitD deficiency and normal VitD levels.

Parameter	Overall n = 208	VitDn n = 82	VitDd n = 126	*p*-Value
GFAP (pg/mL)	2210 (1457, 4450)	2143 (1450, 3626)	3055 (1548, 5045)	0.157
NF-H (pg/mL)	4 (3, 25)	3 (3, 4)	4 (3, 25)	<0.001
S100B (pg/mL)	12 (4, 16)	12 (3, 14)	12 (8, 17)	0.113
UCHL1 (pg/mL)	55 (49, 60)	52 (49, 59)	56 (48, 61)	0.028

VitDn—the group with normal vitamin D3 levels; VitDd—the group with vitamin D3 deficiency; GFAP—glial fibrillary acidic protein; NF-H—neurofilament heavy chains; S100B—calcium-binding protein B, UCHCL1—ubiquitin C-terminal hydrolase L.

## Data Availability

Due to the Personal Data Protection Act, all data are available only upon request from the corresponding author.
